# Circulating Exosomal Gastric Cancer–Associated Long Noncoding RNA1 as a Biomarker for Early Detection and Monitoring Progression of Gastric Cancer: A Multiphase Study

**DOI:** 10.1001/jamasurg.2020.1133

**Published:** 2020-06-10

**Authors:** Xin Guo, Xiaohui Lv, Yi Ru, Fuxing Zhou, Ning Wang, Hongqing Xi, Kecheng Zhang, Jiyang Li, Rongyan Chang, Tianyu Xie, Xinxin Wang, Baohai Li, Yong Chen, Yanling Yang, Lubin Chen, Lin Chen

**Affiliations:** 1 Department of Endoscopic Surgery, Chinese People’s Liberation Army 986th Hospital, Fourth Military Medical University, Shaanxi, China; 2 Department of General Surgery, Chinese People’s Liberation Army General Hospital, Beijing, China; 3 Department of Gynecology and Obstetrics, Xijing Hospital, Fourth Military Medical University, Shaanxi, China; 4 Department of Biochemistry and Molecular Biology, Fourth Military Medical University, Shaanxi, China; 5 Department of Ophthalmology, Chinese People’s Liberation Army 305th Hospital, Nanjing, China; 6 Department of Hepatobiliary Surgery, Xijing Hospital, Fourth Military Medical University, Shaanxi, China

## Abstract

**Importance:**

The gastric cancer (GC)–associated long noncoding RNA1 (lncRNA-GC1) plays an important role in gastric carcinogenesis. However, exosomal lncRNA-GC1 and its potential role in GC are poorly understood.

**Objective:**

To evaluate the diagnostic value of circulating exosomal lncRNA-GC1 for early detection and monitoring progression of GC.

**Design, Setting, and Participants:**

We performed a multiphase investigation of circulating exosomal lncRNA-GC1 for early detection of GC involving consecutive patients with GC (n = 522), patients with gastric precancerous lesions (n = 85), and healthy donor individuals (HDs; n = 219) from December 2016 to February 2019 at Chinese People’s Liberation Army General Hospital, China. LncRNA-GC1 was measured by reverse transcription–polymerase chain reaction by independent researchers who had no access to patients’ information. Receiver operating characteristic curves were used to calculate diagnostic efficiency in comparison between lncRNA-GC1 and 3 traditional biomarkers (carcinoembryonic antigen [CEA], cancer antigen 72-4 [CA72-4], and CA19-9).

**Main Outcomes and Measures:**

Assessment of diagnostic efficiency on the basis of area under curve (AUC), specificity, and sensitivity.

**Results:**

Of the 826 patients included in the study, 508 were men (61.5%), and the median age of all patients was 60 years (range, 28-82 years). In the test phase, lncRNA-GC1 achieved better diagnostic performance than the standard biomarkers CEA, CA72-4, and CA19-9 (AUC = 0.9033) for distinguishing between the patients with GC and HDs. Additionally, exosomal lncRNA-GC1 levels were significantly higher in culture media from GC cells compared with those of normal gastric epithelial cells (*t* = 5.310; *P* = .002). In the verification phase, lncRNA-GC1 retained its diagnostic efficiency in discriminating patients with GC from those with gastric precancerous lesions as well from HDs. Moreover, lncRNA-GC1 exhibited a higher AUC compared with those of CEA, CA72-4, and CA19-9 for early detection of GC with sufficient specificity and sensitivity, especially for patients with GC with negative standard biomarkers. Moreover, the levels of circulating exosomal lncRNA-GC1 were significantly associated with GC from early to advanced stages (HD vs stage I, *t* = 20.98; *P* < .001; stage I vs stage II, *t* = 2.787; *P* = .006; stage II vs stage III, *t* = 4.471; *P* < .001; stage III vs stage IV, *t* = 1.023; *P* = .30), independent of pathological grading and Lauren classification (pathological grading: HD vs G1, *t* = 21.09; *P* < .001; G1 vs G2, *t* = 0.3718; *P* = .71; G2 vs G3, *t* = 0.3598; *P* = .72; Lauren classification: *t* = 24.81; *P* <.001). In the supplemental phase, the levels of circulating exosomal lncRNA-GC1 were consistent with those in GC tissues and cells and were higher compared with those in normal tissues and cells. Furthermore, the levels of circulating lncRNA-GC1 were unchanged after exosomes were treated with RNase and remained constant after prolonged exposure to room temperature or after repeated freezing and thawing (*t* = 1.443; *P* = .39). Total circulating lncRNA-GC1 was nearly all packaged within exosomes rather than a free form in plasma.

**Conclusions and Relevence:**

Circulating exosomal lncRNA-GC1 may serve as a noninvasive biomarker for detecting early-stage GC and for monitoring disease progression. Combining circulating exosomal lncRNA-GC1 detection with endoscopy could improve the early diagnostic rate of GC.

## Key Points

**Questions:**

What role does circulating exosomal long noncoding RNA-GC1 (lncRNA-GC1) play in gastric cancer (GC), and does lncRNA-GC1 exhibit sufficient diagnostic performance for detecting early-stage GC and for monitoring disease progression?

**Findings:**

In this multiphase study involving 826 participants, including patients with GC, patients with gastric precancerous lesions, and healthy donor individuals, circulating exosomal lncRNA-GC1 served as a noninvasive biomarker for detecting early-stage GC and for monitoring disease progression.

**Meaning:**

For patients with gastric cancer, detection of circulating exosomal lncRNA-GC1 may improve the early diagnostic rate and monitor disease progression.

## Teaser

This study evaluates the diagnostic value of circulating exosomal gastric cancer–associated long noncoding RNA1 for early detection and monitoring progression of gastric cancer.

## Introduction

Although its incidence and mortality has decreased, gastric cancer (GC) is the fourth most common cancer and the second leading cause of cancer-related death worldwide, particularly in China.^1,2^ The number of new cases and deaths may compose approximately one-half of the global total.^3,4,5^ The current criterion standard for diagnosing GC is endoscopic biopsy.^6^ However, because of its discomfort to the patient and high cost, screening for early GC (EGC) is a major difficulty in clinical practice, particularly for asymptomatic individuals.^7^ Unfortunately, gastric precursor lesions, such as intestinal metaplasia (IM), chronic atrophic gastritis (CAG), and persistent *Helicobacter pylori* (HP) infection, increase the difficulty of screening for EGC.^8^ Furthermore, the standard serum biomarkers for GC, such as carcinoembryonic antigen (CEA), cancer antigen 72-4 (CA72-4), and CA19-9, achieve a low positive rate.^9,10^ Thus, it is critically important to develop new approaches for diagnosing EGC with high specificity and sensitivity.

Exosomes, 50 to 150 nm in diameter, function in intercellular communications through their secretion by certain cells and are therefore regarded as messengers sent from their cells of origin.^11,12^ Studies show that the GC-associated long noncoding RNA1 (lncRNA-GC1), an RNA Pol II transcript, is a focus of attention.^13^ LncRNA-GC1 functions as a modular scaffold through binding to histone acetyltransferases WDR5 and KAT2A, leading to modifications of histones associated with the target gene *SOD2*, which promotes the progression of GC.^13^ However, the detection of lncRNA-GC1 in circulating exosomes of patients with GC has not, to our knowledge, been reported.

Here we show that lncRNA-GC1 is present in exosomes isolated from serum samples of patients with GC, which led us to systematically investigate the expression of circulating exosomal lncRNA-GC1 in healthy donor individuals (HDs), patients with early GC (EGC), and patients with gastric precancerous lesions. More important, we compared the diagnostic efficiency of lncRNA-GC1 with standard biomarkers, including CEA, CA72-4, and CA19-9. Our ultimate goal was to determine whether circulating exosomal lncRNA-GC1 may serve as highly specific and sensitive biomarker for early detection and monitoring the progression of GC.

## Methods

### Ethical Considerations

This study was approved by the Medical Ethics Committee of the Air Force 986th Military Hospital, Fourth Military Medical University, and the Chinese People’s Liberation Army General Hospital. The study was also in accordance with the International Ethical Guidelines for Biomedical Research Involving Human Subjects (CIOMS)^14^ and the Reporting Recommendations for Tumor Marker Prognostic Studies (REMARK) guidelines.^15^ Patients provided their written informed consent before enrollment. Serum samples were used according to the committees’ regulations.

### Serum and Tissue Samples Collection

The serum and tissue samples collection and storage are shown in eMethods 1 in the Supplement.

### Cell Culture

The obtainment and culture of cell lines were shown in eMethods 2 in the Supplement.

### Exosomes Isolation and Characterization

Cell lines were cultured in 10-cm dishes containing Dulbecco Modified Eagle Medium high-glucose media with 10% exosome-free fetal bovine serum (Gibco). Exosomes isolated from cell culture medium or serum were passed through a 0.22-μm membrane filter (Millipore) and concentrated using ultracentrifugation and identified as previously reported^16^ (eMethods 3 in the Supplement).

### RNA Extraction From Tissues, Exosomes, and Plasma

Total RNA from tissues or exosomes was extracted using a RNeasy Kit (Qiagen) according to the manufacturer’s instructions. Circulating RNA was extracted using a Plasma RNA Kit (Qiagen) according to the manufacturer’s instructions. A fluorometer and an RNA HS Assay Kit (Qiagen) were used to determine the purities and concentrations of the RNA preparations.

### Real-time Polymerase Chain Reaction and Cutoff Value Selection

The isolation and synthetization of RNA and amplification of DNA were shown in eMethods 4 in the Supplement. The optimal cutoff value of lncRNA-GC1 was generated based on the verification cohort and determined when the Youden index (Youden index = specificity + sensitivity − 1) was the highest. Thus, the optimal cutoff value was 5.200 with the highest Youden index of 0.6974.

### Carcinoembryonic Antigen, CA72-4, and CA19-9 Assays

The serum levels of CEA, CA72-4, and CA19-9 were measured using Elecsys-electrochemical Immune Assays (Roche). The cutoff values of CEA, CA72-4, and CA19-9 were 5 ng/mL, 5.3 U/mL, and 27 U/mL, respectively.

### Statistical Analysis

Data were analyzed and displayed using SPSS, version 18.0 (IBM), and GraphPad Prism 8.0 software (GraphPad). Data are shown as the form of mean (SD). The *t* test was used to analyze the differences between the mean values of 2 groups. Receiver operating characteristic (ROC) curves were plotted to evaluate diagnostic efficiency. The significance of the correlations between 2 variables was analyzed using the Pearson correlation test. Clinical variables were analyzed using Pearson χ^2^ test. A 2-tailed *P* value less than .05 indicates a significant difference.

## Results

### Study Design

**Figure 1.**
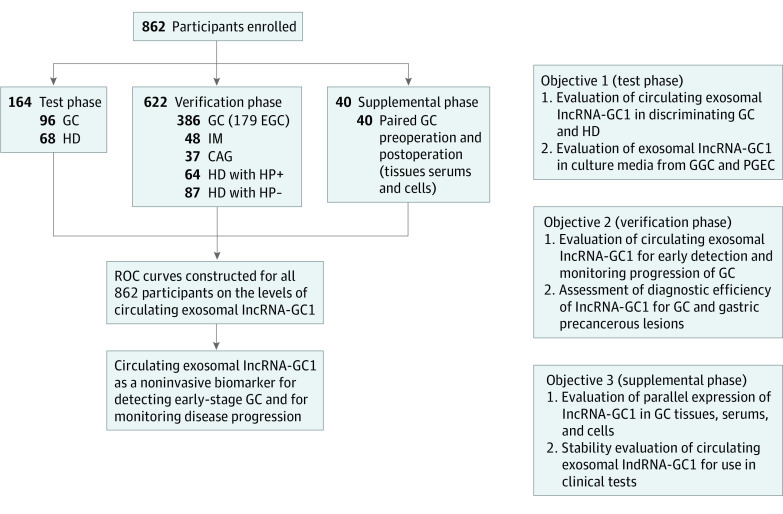
Study Design The levels of circulating exosomal gastric cancer–associated long noncoding RNA1 (lncRNA-GC1) were analyzed in 3 different groups of patients. CAG indicates chronic atrophic gastritis; EGC, early gastric cancer; HD, healthy donor individual; HP, *Helicobacter pylori*; IM, intestinal metaplasia.

Our goal was to prove whether circulating exosomal lncRNA-GC1 serves as a biomarker for the early detection of GC and for monitoring disease progression. To this end, we conducted a 3-phase study. The study design is illustrated in Figure 1. The clinicopathologic characteristics of patients in the 3 phases are presented in eTable 1 in the Supplement.

### Levels of Exosomal lncRNA-GC1 in the Test Phase and in GC Cell Lines

Exosomes were isolated from patients’ sera or the conditioned culture media of GC cells (GGCs). Electron microscopy was used to confirm the presence of exosomes (eFigure 1A in the Supplement). NanoSight particle tracking indicated that the diameter of exosomes ranged from 80 to 120 nm (eFigure 1B in the Supplement). Western blotting detected the exosome-specific markers CD9 and CD63 but not the negative control (tubulin) (eFigure 1C in the Supplement).

**Figure 2.**
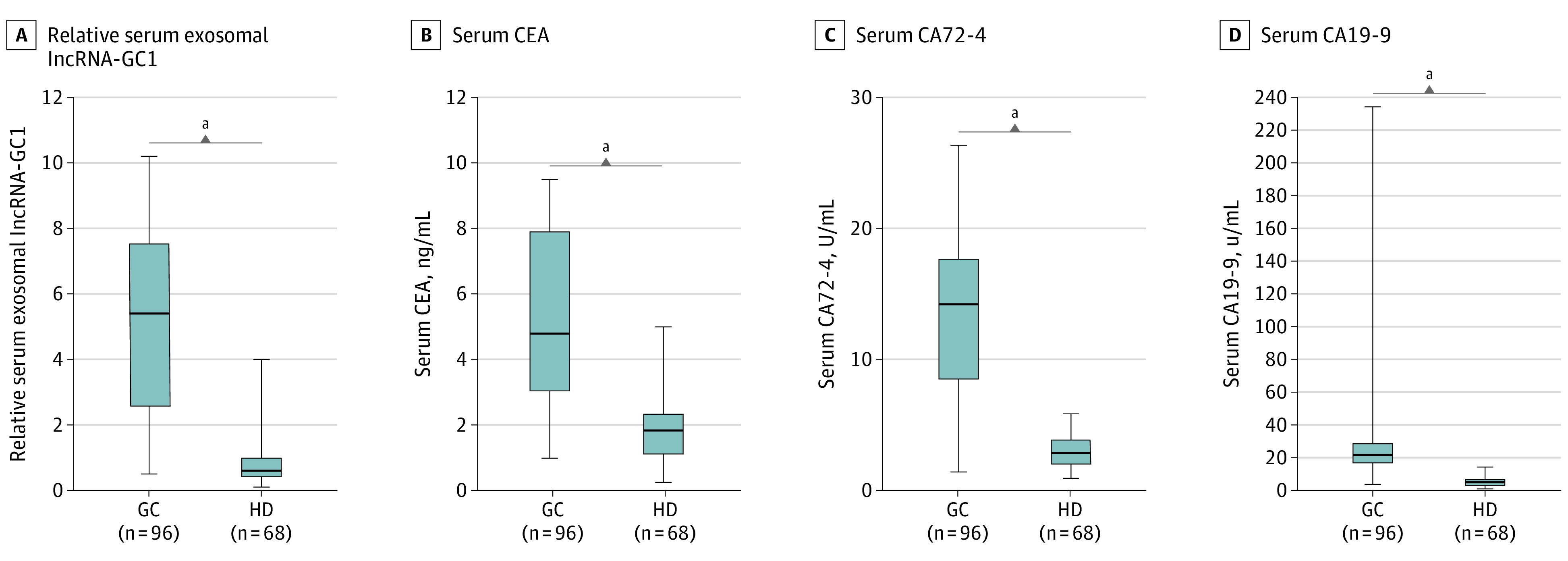
Expression Levels of Gastric Cancer (GC)–Associated Long Noncoding RNA1 (lncRNA-GC1), Carcinoembryonic Antigen (CEA), Cancer Antigen 72-4 (CA72-4), and CA19-9 in the Test Phase A, The relative levels of circulating exosomal lncRNA-GC1 in patients with GC (n = 96) and healthy donor individuals (HDs) (n = 68). B-D, Levels of the standard biomarkers CEA (B), CA72-4 (C), and CA19-9 (D) for patients with GC and HDs. The results are presented as mean (SD). ^a^*P* < .001.

During the test phase, the circulating levels of exosomal lncRNA-GC1 and serum CEA were significantly higher in patients with GC (n = 96) compared with those of the HDs (n = 68; *P* < .001) (Figure 2A and B). Similarly, the serum levels of CA72-4 and CA19-9 of patients with GC were higher compared with those of the HDs (*t* = 13.15 and *t* = 5.624; *P* < .001) (Figure 2C and D). Moreover, the area under the curve (AUC) values of circulating exosomal lncRNA-GC1 were higher compared with those of CEA, CA72-4, and CA19-9 (0.9033 vs 0.5987, 0.6816, and 0.6482, respectively) for distinguishing between patients with GC and HDs (eFigure 2A and eTable 2 in the Supplement). The sensitivity and specificity of lncRNA-GC1 were also higher than those of CEA, CA72-4, and CA19-9 (eTable 2 in the Supplement). Although the levels of exosomal lncRNA-GC1 and the 3 standard markers were all higher in patients with GC compared with those of HDs, lncRNA-GC1 had the highest AUC for distinguishing between them.

Furthermore, the exosomal levels of lncRNA-GC1 in culture media from 10 different GGCs were significantly higher compared with those from *GES-1* cells and primary gastric epithelial cells (PGECs) (*t* = 5.272 and *t* = 5.310; both *P* < .001) (eFigure 2B in the Supplement). Together, these results indicate that compared with standard GC markers, circulating exosomal lncRNA-GC1 may serve as a GC-specific lncRNA, with a sufficient AUC for distinguishing it from HDs.

### Verification of Circulating Exosomal lncRNA-GC1 in EGC

**Figure 3.**
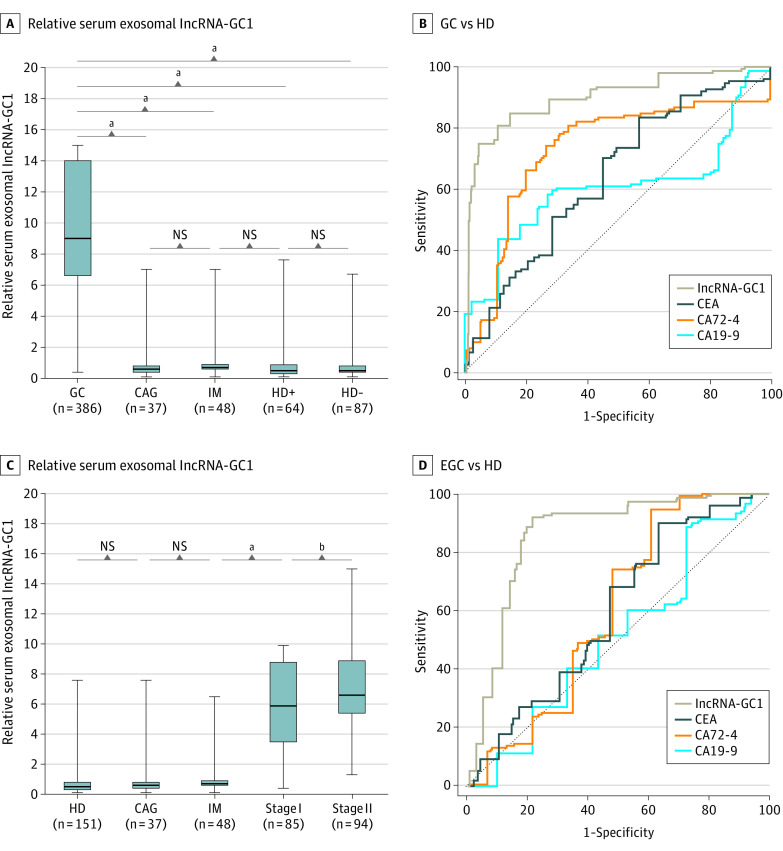
Expression Levels and Diagnostic Values of Gastric Cancer (GC)–Associated Long Noncoding RNA1 (lncRNA-GC1), Carcinoembryonic Antigen (CEA), Cancer Antigen 72-4 (CA72-4), and CA19-9 in the Verification Phase A, The relative levels of circulating exosomal lncRNA-GC1 in patients with GC (n = 386), patients with chronic atrophic gastritis (CAG) (n = 37), patients with intestinal metaplasia (IM) (n = 48), healthy donor individuals with positive *Helicobacter pylori* (HD^+^) (n = 64), and healthy donor individuals (HDs) with negative *H pylori* (HD^-^) (n = 87). B, The receiver operating characteristic curves of lncRNA-GC1, CEA, CA72-4, and CA19-9 in distinguishing GC from HD. C, The relative levels of circulating exosomal lncRNA-GC1 in stage I GC (n = 85), stage II GC (n = 94), CAG (n = 37), IM (n = 48), and HD (n = 151). D, The receiver operating characteristic curves of lncRNA-GC1, CEA, CA72-4, and CA19-9 used to distinguish patients with early GC (EGC) from HDs. Early GC was defined as stages I and II GC. The results are presented as mean (SD). NS indicates not significant. ^a^*P* < .001. ^b^*P* < .01.

In the verification phase, the circulating exosomal lncRNA-GC1 levels in patients with GC were significantly higher compared with those of patients with CAG, patients with IM, patients who were HP positive, and patients who were HP negative (*t* = 14.43, *t* = 16.38, *t* = 18.94, and *t* = 22.62; all *P* < .001) (Figure 3A). There was not a significant difference between the 4 control groups (CAG, IM, HP-positive, and HP-negative). Furthermore, the levels of CEA, CA72-4, and CA19-9 were higher in patients with GC compared with those of patients with CAG, patients with IM, patients who were HP positive, and patients who were HP negative (CEA: GC vs CAG, *t* = 2.949; GC vs IM, *t* = 5.077; GC vs HD positive, *t* = 6.86; GC vs HD negative, *t* = 9.423; all *P < *.001; CA72-4: GC vs CAG, *t* = 7.047; GC VS IM, *t* = 7.300; GC vs HD positive, *t* = 10.11; GC vs HD negative; *t* = 12.25; all *P* < .001; CA19-9: GC vs CAG, *t* = 4.198; GC vs IM, *t* = 4.605; GC vs HD positive, *t* = 5.136; GC vs HD negative, *t* = 6.234; all *P* < .001) (eFigure 3A-C in the Supplement). However, the AUCs, sensitivity, and specificity of circulating exosomal lncRNA-GC1 were all higher compared with those of CEA, CA72-4, and CA19-9 for distinguishing patients with GC from HDs and patients with gastric precancerous lesions (patients with CAG and patients with IM) (Figure 3B; eFigure 4A-B and eTable 2 in the Supplement).

When we further evaluated the diagnostic value of lncRNA-GC1 for early detection of GC, we found that the levels of circulating exosomal lncRNA-GC1 in patients with stages I or II GC were significantly upregulated compared with those in the HDs and patients with CAG or IM (stage I GC vs HDs, *t* = 21.15; stage I GC vs CAG, *t* = 10.93; stage I GC vs IM, *t* = 12.63; stage II GC vs HDs, *t* = 23.82; stage I GC vs CAG, *t* = 12.11; stage I GC vs IM, *t* = 13.93; all *P* < .001) (Figure 3C). However, the levels of CEA, CA72-4, and CA19-9 in patients with stage I or II GC were similar to those of the HDs (eFigure 5A-C in the Supplement). The AUCs, sensitivity, and specificity of circulating exosomal lncRNA-GC1 were all higher compared with those of CEA, CA72-4, and CA19-9 for distinguishing patients with early GC from HDs as well as from patients with CAG or IM (Figure 3D; eFigure 4C-D and eTable 2 in the Supplement).

**Figure 4.**
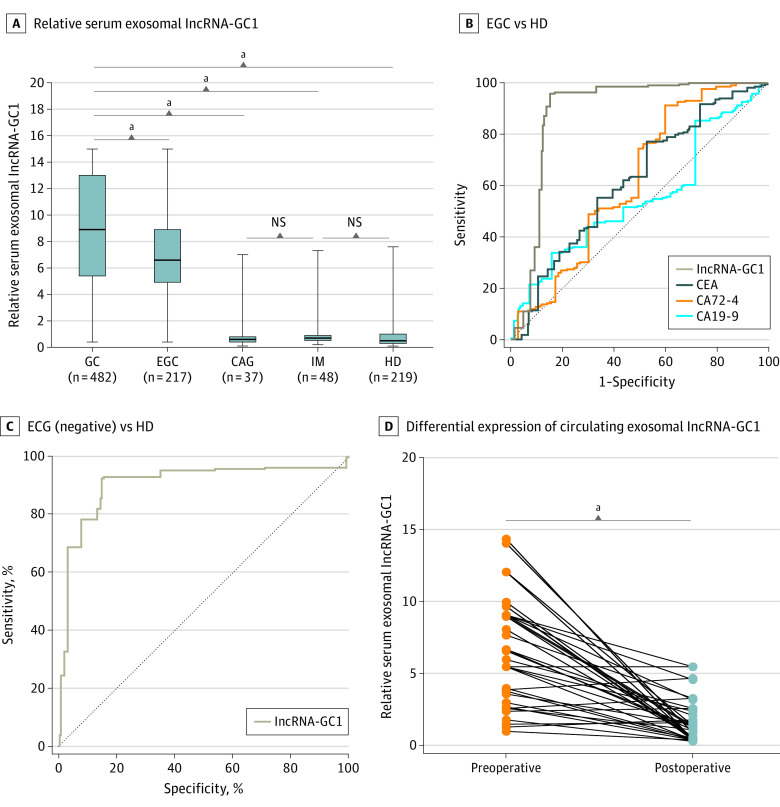
Expression Levels and Diagnostic Values of Gastric Cancer (GC)–Associated Long Noncoding RNA1 (lncRNA-GC1), Carcinoembryonic Antigen (CEA), Cancer Antigen 72-4 (CA72-4), and CA19-9 in the Total Phase (Test and Verification) A, The relative levels of circulating exosomal lncRNA-GC1 in patients with GC (n = 482), patients with early gastric cancer (EGC) (n = 217), patients with chronic atrophic gastritis (CAG) (n = 37), patients with intestinal metaplasia (IM) (n = 48), and healthy donor individuals (HDs) (n = 219). B, Receiver operating characteristic curves of lncRNA-GC1, CEA, CA72-4, and CA19-9 in distinguishing EGC from HD. C, Receiver operating characteristic curves of lncRNA-GC1 in distinguishing patients with EGC with negative status of CEA, CA72-4, and CA19-9 from HD. D, Differential expression of circulating exosomal lncRNA-GC1 between patients with GC before surgery and 5 days after surgery in the supplemental phase. The results are presented as mean (SD). ^a^*P* < .001.

When we pulled patients with GC in the test and verification phases together, the levels of circulating exosomal lncRNA-GC1 for GC as well as for EGC remained higher compared with those in the HDs and patients with precancerous controls (GC vs EGC, *t* = 6.972; *P* < .001; GC vs CAG, *t* = 11.96; *P* < .001; GC vs IM, *t* = 13.61; *P* < .001; GC vs HD, *t* = 29.38; *P* < .001; CAG vs IM, *t* = 0.047; *P* = .96; IM vs HD, *t* = 1.019; *P* = .30) (Figure 4A). The levels of the 3 standard biomarkers still failed to distinguish EGC from HD (eFigure 6A-C in the Supplement). Receiver operating characteristic curves indicated that lncRNA-GC1 had higher AUCs, sensitivity, and specificity compared with CEA, CA72-4, and CA19-9 for distinguishing patients with EGC from HDs as well as from patients with CAG or IM (Figure 4B; eFigure 7A-B and eTable 2 in the Supplement). Furthermore, lncRNA-GC1 retained its high diagnostic efficiency for differentiating GC and especially EGC, with negative status of CEA, CA72-4, and CA19-9, from HDs (Figure 4C). Together, these results strongly indicate the significant diagnostic value of circulating exosomal lncRNA-GC1 levels for the detection of early GC.

### Use of Exosomal lncRNA-GC1 Levels in Monitoring the Progression of GC

**Figure 5.**
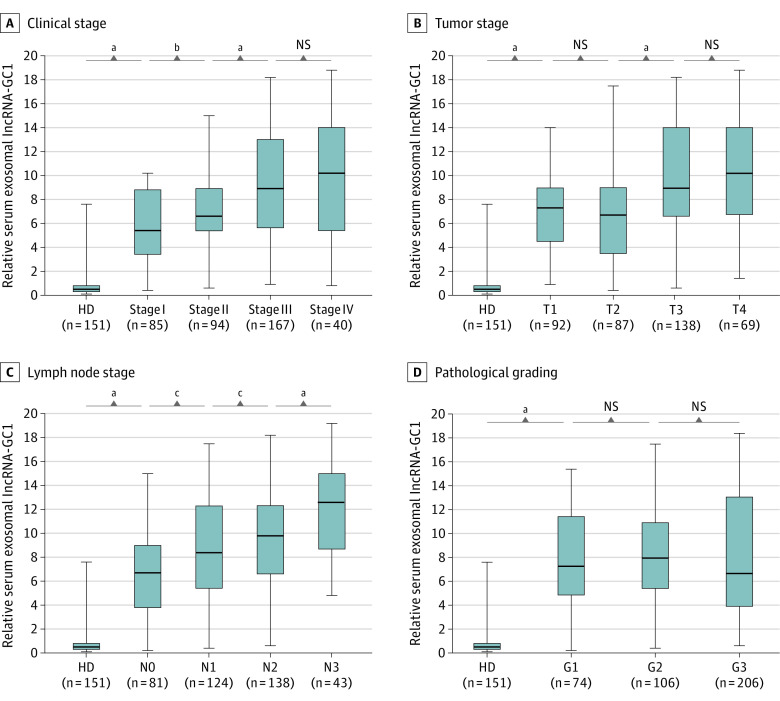
Differential Expression of Gastric Cancer (GC)–Associated Long Noncoding RNA1 (lncRNA-GC1) in the Verification and Supplemental Phases A–D, The relative levels of circulating exosomal lncRNA-GC1 in subgroups of patients with GC according to clinical stage (A), tumor stage (B), lymph node stage (C), and pathological grading (D) in the verification phase. The results are presented as mean (SD). T indicates tumor; G, pathological grade; N, lymph node; NS, not significant. ^a^*P* < .001. ^b^*P* < .01. ^c^*P* < .05.

To determine whether lncRNA-GC1 levels were associated with the progression of GC, in the verification phase, we determined the levels of circulating exosomal lncRNA-GC1 in patients with different TNM stages and differentiation grades. The levels of circulating exosomal lncRNA-GC1 increased incrementally with clinical stages I to IV, and there were significant differences between the 4 clinical stages compared with those of HDs (HD vs stage I, *t* = 20.98; *P* < .001; stage I vs stage II, *t* = 2.787; *P* = .006; stage II vs stage III, *t* = 4.471; *P* < .001) (Figure 5A).

Next, the levels of circulating exosomal lncRNA-GC1 were all increased as a function of T and N stages compared with those of HDs and incrementally increased from stages T1 to T4 and N0 to N3 (Figure 5B and C). The levels of circulating exosomal lncRNA-GC1 remained higher as a function of pathological grade compared with those of HDs. However, there was no significance between GC grades G1 to G3 (Figure 5D). The levels of circulating exosomal lncRNA-GC1 in the intestinal or diffuse types (Lauren classification) of GC were higher compared with those of HDs. However, there was no significant difference between the intestinal and diffuse type (eFigure 7C in the Supplement).

To investigate the temporal changes in levels of exosomal lncRNA-GC1 during primary diagnosis, extending to the time after surgery, we next analyzed 40 paired serum samples acquired from patients with GC before and 5 days after gastrectomy in the supplemental phase. Thirty-five patients showed a significant decrease in the levels of circulating exosomal lncRNA-GC1 in postoperative serum compared with ones in the preoperative serum (*t* = 7.951; *P < *.001) (Figure 4D). Together, these results revealed that the levels of circulating exosomal lncRNA-GC1 that were strictly correlated with tumor burden significantly increased with the progression of GC from early to advanced stages and were independent of tumor differentiation and Lauren classification.

### Parallel Expression of lncRNA-GC1 in Patients With GC and GGCs

Although the levels of circulating exosomal lncRNA-GC1 were high in patients with GC, it was important to determine whether they were consistent with those of GC tumor tissues. Therefore, we analyzed the levels of lncRNA-GC1 in 40 GC tissues as well as in the adjacent noncancerous tissues as well as in serum exosomes from the same patients. We detected higher levels of lncRNA-GC1 in cancerous tissues and exosomes compared with those of the corresponding adjacent noncancerous tissues in 36 of 40 GC tissues (eFigure 8A in the Supplement). Furthermore, 10 GGCs expressed higher levels of lncRNA-GC1 compared with those of GES-1 cells and PGECs (eFigure 8B in the Supplement), and the exosomal levels of lncRNA-GC1 in culture media of the 10 GGCs were higher compared with those of GES-1 cells and PGECs. Together, these results indicate that the exosomal levels of lncRNA-GC1 were consistent with those of cancerous GC tissues and served as a GC-specific lncRNA.

### Stability of Circulating Exosomal lncRNA-GC1 in Patients With GC

The instability of lncRNAs in serum remains a major limitation for clinical application. Thus, we randomly collected 15 GC serum samples to investigate the stability of lncRNA-GC1. We found that the levels of lncRNA-GC1 in sera were constant after treatment with RNase (*t* = 2.657; *P = *.39) (eFigure 9A in the Supplement). Furthermore, prolonged exposure to room temperature and repeated freezing and thawing had no influence on the serum levels of lncRNA-GC1 (eFigure 9B and C in the Supplement). Moreover, the circulating levels of lncRNA-GC1 were abundant in exosomes compared with those of exosome-depleted sera (eFigure 9D in the Supplement). Finally, we evaluated the association between serum exosomal lncRNA-GC1 levels and total serum lncRNA-GC1 levels. The exosomal serum levels of lncRNA-GC1 was associated with total serum levels of lncRNA-GC1 (eFigure 9E in the Supplement). Together, these results suggest that the circulating levels of lncRNA-GC1 were nearly all encapsulated in exosomes, which were thereby protected, conferring sufficient stability of lncRNA-GC1 for use in clinical tests.

## Discussion

Late detection is a major reason for the poor prognosis of patients with GC. For example, the proportion of patients diagnosed as having EGC is as low as 9% in China.^17^ The survival rate of patients with EGC ranges from 60% to 80% compared with 15% to 24% of patients with advanced GC.^18^ It is therefore imperative to develop novel, relatively noninvasive approaches to improve early diagnosis of GC. To address this formidable challenge, we conducted a 3-phase study. In the first (test) phase, the circulating exosomal lncRNA-GC1 levels had higher sensitivity and specificity compared with CEA, CA72-4, and CA19-9, which failed to distinguish between patients with GC and HD.

In the second phase (verification), the CEA, CA72-4, and CA19-9 levels failed to distinguish between EGC and HD, consistent with the results of other studies.^19,20,21^ For gastric precancerous lesions, these biomarkers failed to distinguish between patients with EGC, CAG, or IM. In contrast, lncRNA-GC1 levels achieved better diagnostic efficiency. To our knowledge, normal GECs do not undergo rapid malignant transformation to GC, which is a stepwise process. Malignant progression involves the major steps as follows: chronic superficial gastritis develops into chronic atrophic gastritis, which in turn progresses to intestinal metaplasia and dysplasia, and finally generates the malignant phenotype of GC.^22,23^ Thus, it is essential to screen healthy individuals as well as those with precancerous lesions.

More importantly, the level of lncRNA-GC1 retained its diagnostic efficiency for distinguishing between patients with EGC and those with gastric precancerous lesions. Furthermore, the levels of circulating exosomal lncRNA-GC1 incrementally increased from EGC to advanced GC, suggesting that lncRNA-GC1 levels may accurately reflect the progression of GC. Thus, we believe our findings contribute compelling preliminary evidence indicating that circulating exosomal lncRNA-GC1 levels can serve as a novel biomarker for early detection and monitoring the progression of GC.

In the supplemental phase, we asked whether the circulating levels of exosomal lncRNA-GC1 remained constant, which is an important criterion for application as a routine clinical assay. Fortunately, the levels of circulating exosomal lncRNA-GC1 remained stable and were protected by their encasement in exosomes. Together, these results further confirm that lncRNA-GC1 is packaged in exosomes as a GC-specific RNA. Moreover, our primary concern was the generalizability of lncRNA-GC1 measurement. For now, the main restrictions are the stable acquisition of exosomes and the dedicated isolation of exosomes, making it difficult in routine clinical practice. Additionally, the turnaround time was 4 days and the costs are nearly $120, which we believe will be lower after integrative optimization.

In clinical practice, neoadjuvant chemotherapy has been increasingly used in the treatment of GC. For those patients with locally advanced GC, neoadjuvant chemotherapy may provide a potential opportunity of curative surgery. However, the effects of neoadjuvant chemotherapy are mainly estimated by computed tomographic scan. Thus, we have wondered whether the levels of lncRNA-GC1 could measure the effects of neoadjuvant chemotherapy. In a cohort of 49 patients with GC who underwent neoadjuvant chemotherapy, 37 (75.5%) exhibited decreased levels of lncRNA-GC1 (data not shown). These results shed new light on the measurement of neoadjuvant chemotherapy; however, these should be verified in a larger cohort.

## Limitations

To our knowledge, this is the first study to systematically investigate the diagnostic efficiency of circulating exosomal lncRNA-GC1 levels for GC. Although we demonstrate here the potential ability of lncRNA-GC1 for early detection and for monitoring the progression of GC, we note limitations to our study. First, we enrolled a relatively small number of patients and healthy control individuals, and the patients with GC were pathologically diagnosed before the confirmation of high levels of lncRNA-GC1. Our ultimate goal is to measure the levels of circulating exosomal lncRNA-GC1 in asymptomatic individuals before they are diagnosed as having GC. Second, we conducted a retrospective, single-center, cross-sectional study that may have introduced unavoidable selection bias. To further confirm the diagnostic efficiency of lncRNA-GC1, a prospective and multicenter study will be required. The follow-up analysis is ongoing. Finally, the diagnostic efficacy was not compared between lncRNA-GC1 and endoscopy. For now, the narrow-band imaging (NBI) of endoscopy is widely used for screening EGC and shows promising results. It is reported that the sensitivity and specificity of NBI were as high as 93% and 95%, which were higher than those of lncRNA-GC1.^24^ Thus, we believe that lncRNA-GC1 may complement endoscopy and provide sufficient diagnostic efficacy for screening EGC. However, fewer than 40% of patients with GC received NBI endoscopy in this study, making it difficult to compare lncRNA-GC1 with NBI endoscopy. Our future studies will attempt to test this comparison.

## Conclusions

To our knowledge, this study is the first to establish that the high levels of circulating lncRNA-GC1 reside in exosomes specifically associated with GC. Furthermore, the protection of lncRNA-GC1 by exosomes confers stability in the circulation, making possible reproducible detection. Additionally, lncRNA-GC1 had better performance in distinguishing GC from precancerous lesions. More important, circulating exosomal lncRNA-GC1 exhibited great promise as a biomarker for the early detection of GC and for monitoring disease progression.

## Notes

Supplement. eFigure 1. Isolation and characterization of exosomes. eFigure 2. The diagnostic values of lncRNA-GC1, CEA, CA72-4 and CA19-9 in the test phase and cell lines. eFigure 3. The levels of CEA, CA72-4, and CA19-9 in the verification phase. eFigure 4. The diagnostic values of lncRNA-GC1, CEA, CA72-4, and CA19-9 in the verification phase. eFigure 5. The levels of CEA, CA72-4, and CA19-9 in EGC. eFigure 6. The levels of CEA, CA72-4, and CA19-9 in total phase. eFigure 7. The diagnostic values and expression levels of lncRNA-GC1, CEA, CA72-4, and CA19-9. eFigure 8. Expression of lncRNA-GC1 in patients with GC and in GGCs. eFigure 9. The stability of circulating lncRNA-GC1 in patients with GC. eTable 1. Clinical characteristics of individuals in test, verification and supplemental phases. eTable 2. Results of ROC curves for circulating lncRNA-GC1, CEA, CA72-4 and CA19-9. **eMethods 1.** Serum and tissue samples collection **eMethods 2.** Cell culture **eMethods 3.** Identifications of exosomes. **eMethods 4.** Real-time PCR.
