# Supplementary material for: Circulating Exosomal Gastric Cancer–Associated Long Noncoding RNA1 as a Biomarker for Early Detection and Monitoring Progression of Gastric Cancer: A Multiphase Study
Source: JAMA Surg. 2020 Jun 10;155(7):572–9. doi: 10.1001/jamasurg.2020.1133 (PMC7287948; doi:10.1001/jamasurg.2020.1133)
Supplement: Supplement. — eFigure 1. Isolation and characterization of exosomes. eFigure 2. The diagnostic values of lncRNA-GC1, CEA, CA72-4 and CA19-9 in the test phase and cell lines. eFigure 3. The levels of CEA, CA72-4, and CA19-9 in the verification phase. eFigure 4. The diagnostic values of lncRNA-GC1, CEA, CA72-4, and CA19-9 in the verification phase. eFigure 5. The levels of CEA, CA72-4, and CA19-9 in EGC. eFigure 6. The levels of CEA, CA72-4, and CA19-9 in total phase. eFigure 7. The diagnostic values and expression levels of lncRNA-GC1, CEA, CA72-4, and CA19-9. eFigure 8. Expression of lncRNA-GC1 in patients with GC and in GGCs. eFigure 9. The stability of circulating lncRNA-GC1 in patients with GC. eTable 1. Clinical characteristics of individuals in test, verification and supplemental phases. eTable 2. Results of ROC curves for circulating lncRNA-GC1, CEA, CA72-4 and CA19-9. eMethods 1. Serum and tissue samples collection eMethods 2. Cell culture eMethods 3. Identifications of exosomes. eMethods 4. Real-time PCR. [file jamasurg-155-572-s001.pdf]

Supplementary Online Content

Guo X, Lv X, Ru Y, et al. Circulating exosomal gastric cancer–associated long noncoding RNA1 as a biomarker for early detection and monitoring progression of gastric cancer: a multiphase study. Published online June 10, 2020. *JAMA Surg*. doi:10.1001/jamasurg.2020.1133

eFigure 1. Isolation and characterization of exosomes.
eFigure 2. The diagnostic values of lncRNA-GC1, CEA, CA72-4 and CA19-9 in the test phase and cell lines.
eFigure 3. The levels of CEA, CA72-4, and CA19-9 in the verification phase.
eFigure 4. The diagnostic values of lncRNA-GC1, CEA, CA72-4, and CA19-9 in the verification phase.
eFigure 5. The levels of CEA, CA72-4, and CA19-9 in EGC.
eFigure 6. The levels of CEA, CA72-4, and CA19-9 in total phase.
eFigure 7. The diagnostic values and expression levels of lncRNA-GC1, CEA, CA72-4, and CA19-9.
eFigure 8. Expression of lncRNA-GC1 in patients with GC and in GGCs.
eFigure 9. The stability of circulating lncRNA-GC1 in patients with GC.
eTable 1. Clinical characteristics of individuals in test, verification and supplemental phases.
eTable 2. Results of ROC curves for circulating lncRNA-GC1, CEA, CA72-4 and CA19-9.
eMethods 1. Serum and tissue samples collection
eMethods 2. Cell culture
eMethods 3. Identifications of exosomes.
eMethods 4. Real-time PCR.

This supplementary material has been provided by the authors to give readers additional information about their work.

© 2020 Guo X et al. *JAMA Surg.*

eFigure 1. Isolation and characterization of exosomes.

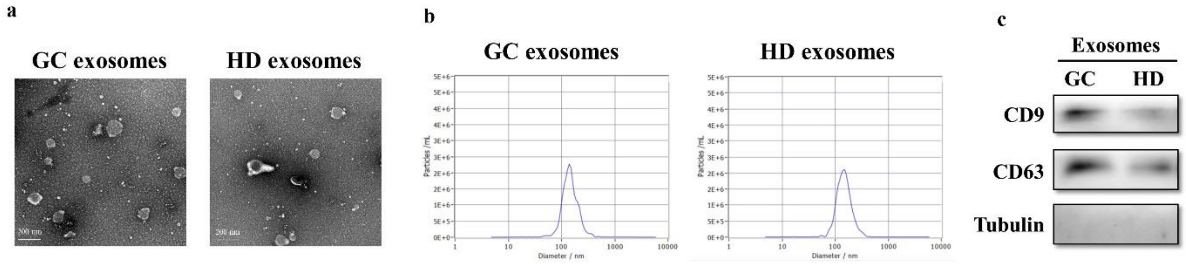

(a) Exosomes were observed using electron microscopy, which revealed characteristic vesicular structures. Scale bar = 100 nm. **(b)** The size distributions of exosomes were analyzed using NanoSight particle-tracking analysis, which revealed particle sizes ranging from 80 nm to 120 nm. **(c)** Western blot analysis detected the exosomal markers CD9 and CD63 but not tubulin.

© 2020 Guo X et al. *JAMA Surg.*

eFigure 2. The diagnostic values of lncRNA-GC1, CEA, CA72-4 and CA19-9 in the test phase and cell lines.

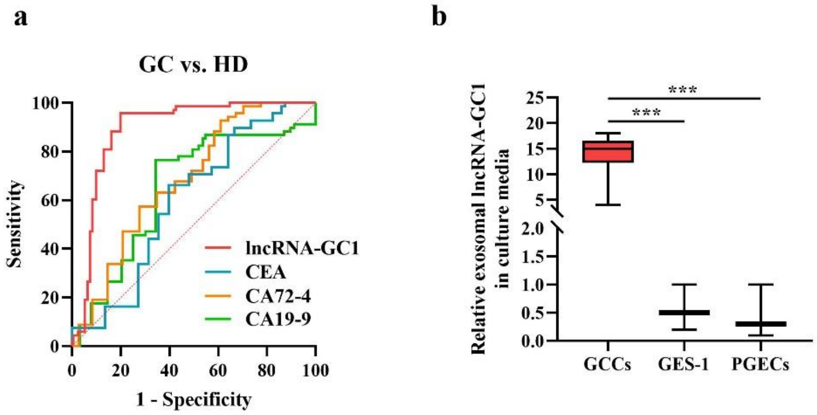

(a) The ROC curves of lncRNA-GC1, CEA, CA72-4, and CA19-9 for distinguishing patients with GC from HDs. **(b)** The relative levels of exosomal lncRNA-GC1 in GGCs (n = 10) and normal gastric epithelial cells. The results are presented as the mean ± SD. *** P<0.001.

eFigure 3. The levels of CEA, CA72-4, and CA19-9 in patients with early-stage GC and precancerous lesions.

© 2020 Guo X et al. *JAMA Surg.*

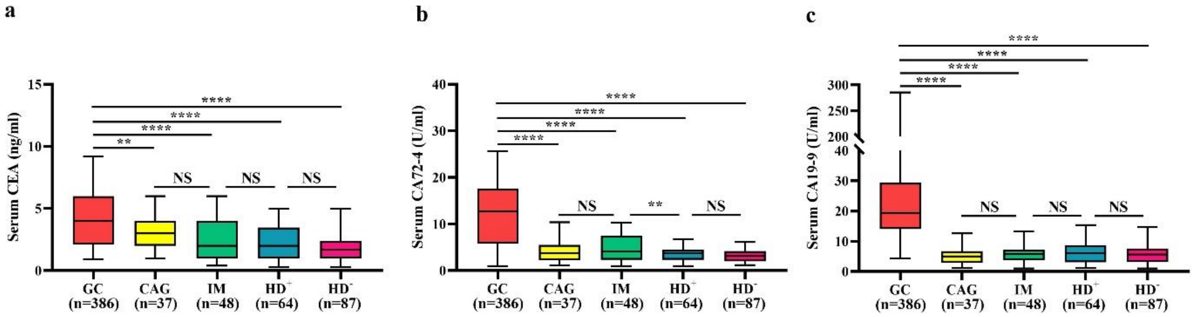

The levels of CEA **(a)**, CA72-4 **(b)**, and CA19-9 **(c)** in the verification phase including patients with GC (n = 386), patients with CAG (n = 37), patients with IM (n = 48), HDs$^+$ (n = 64) and HDs$^-$ (n = 87). The results are presented as the mean ± SD. ** P<0.01; **** P<0.0001.

eFigure 4. The diagnostic values of lncRNA-GC1, CEA, CA72-4, and CA19-9 in the verification phase.

© 2020 Guo X et al. *JAMA Surg.*

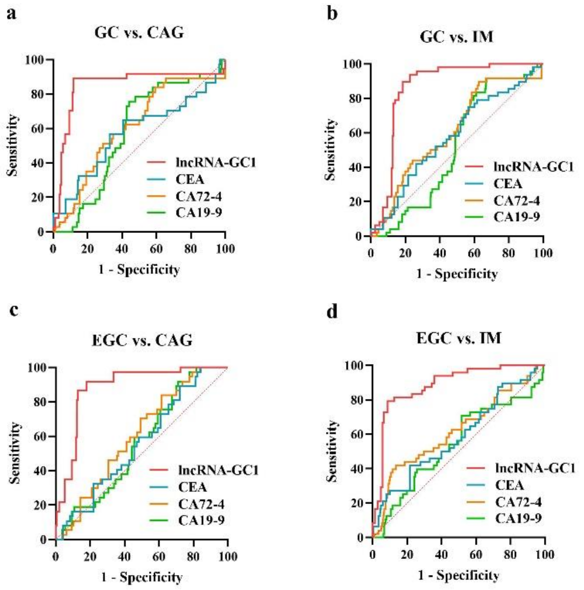

(a and b) The ROC curves of lncRNA-GC1, CEA, CA72-4, and CA19-9 in distinguishing GC from CAG **(a)** and GC from IM **(b)**. **(c and d)** The ROC curves of lncRNA-GC1, CEA, CA72-4, and CA19-9 used to distinguish patients with EGC from those with CAG **(c)** and patients with EGC from those with IM **(d)**. EGC was defined as stages I and II GC. The results are presented as the mean ± SD. NS, not significant; ** P<0.01; *** P<0.001; **** P<0.0001.

eFigure 5. The levels of CEA, CA72-4, and CA19-9 in EGC.

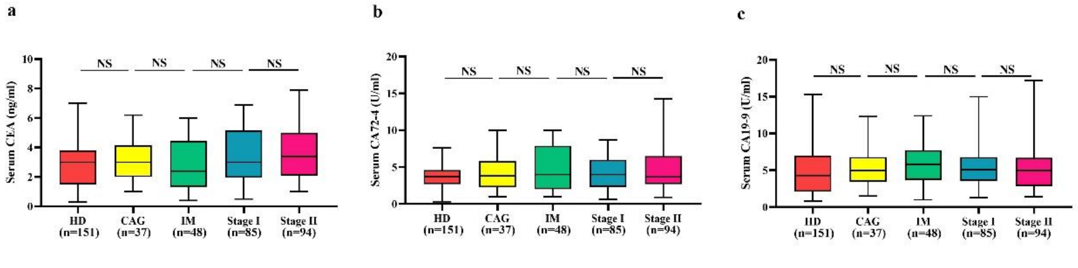

The levels of CEA **(a)**, CA72-4 **(b)**, and CA19-9 **(c)** in patients with stages I (n = 85) and II GC (n = 94), patients with CAG (n = 37), patients with IM (n = 48), and HDs (n = 151) in the verification phase. The results are presented as the mean ± SD. NS, not

© 2020 Guo X et al. *JAMA Surg.*

significant.

eFigure 6. The levels of CEA, CA72-4, and CA19-9 in total phase.

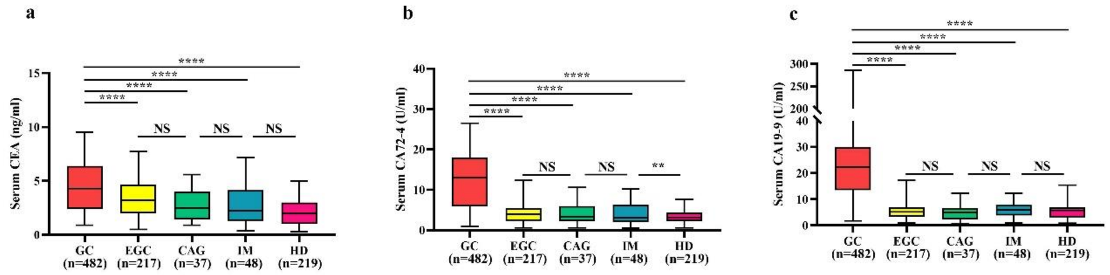

The levels of CEA **(b)**, CA72-4 **(c)**, and CA19-9 **(d)** in the total phase (test and verification phases) including patients with GC (n = 482), patients with EGC (n = 217), patients with CAG (n = 37), patients with IM (n = 48) and HDs (n = 219). The results are presented as the mean ± SD. NS, not significant; ** P<0.01; *** P<0.001; **** P<0.0001.

eFigure 7. The diagnostic values and expression levels of lncRNA-GC1, CEA, CA72-

4, and CA19-9.

a

b

c

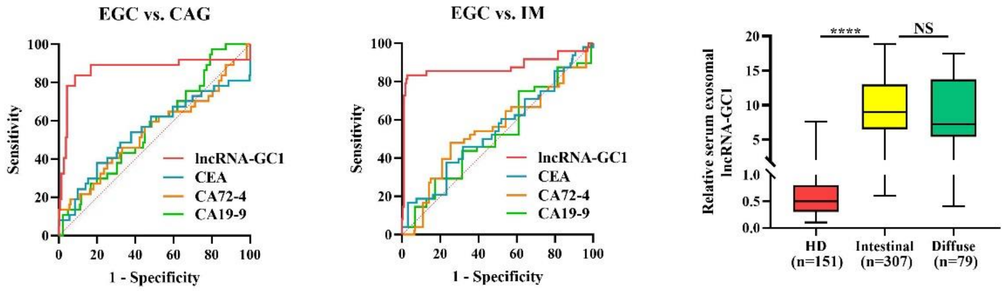

(a and b) The ROC curves of lncRNA-GC1, CEA, CA72-4, and CA19-9 in distinguishing EGC from CAG **(a)** and EGC from IM **(b)**. **(c)** The relative levels of circulating exosomal lncRNA-GC1 in subgroups of patients with GC according to Lauren's Classification **(c)**. The results are presented as the mean ± SD. NS, not significant; ** P<0.01; *** P<0.001; **** P<0.0001.

eFigure 8. Expression of lncRNA-GC1 in patients with GC and in GGCs.

© 2020 Guo X et al. *JAMA Surg.*

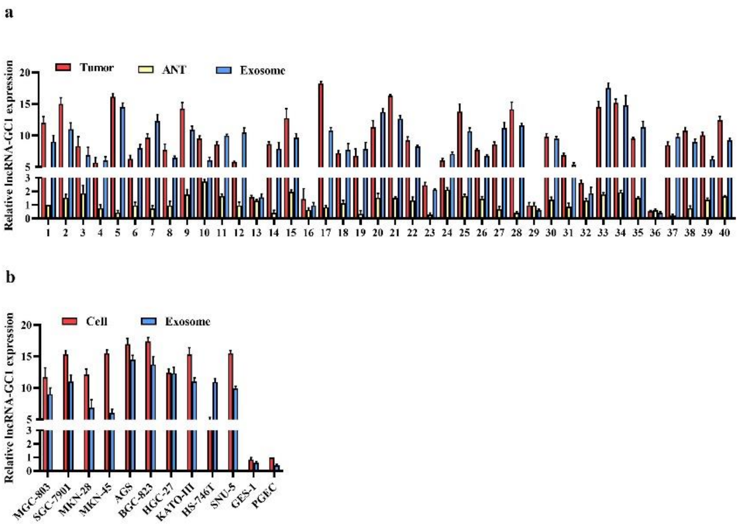

(a) The levels of lncRNA-GC1 in paired tumor and ANT tissues and the corresponding exosomes. **(b)** The expression levels of lncRNA-GC1 in GGCs and normal gastric epithelial cells. The results are presented as the mean ± SD.

eFigure 9. The stability of circulating lncRNA-GC1 in patients with GC.

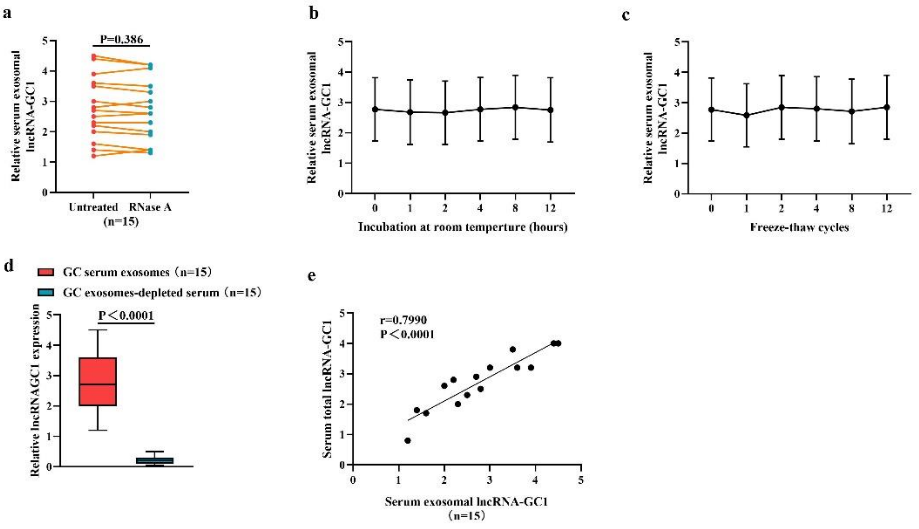

Relative serum exosomal levels of lncRNA-GC1 in patients with GC (n = 15) when

© 2020 Guo X et al. *JAMA Surg.*

exosomes were **(a)** treated with or without RNase A (5 µg/ml) for 30 min, **(b)** subjected to prolonged incubation to room temperature and **(c)** subjected to with repeated freezing and thawing. **(d)** Relative levels of lncRNA-GC1 in exosomes and exosome-depleted sera of patients with GC (n = 15). **(e)** Positive correlations between serum exosomal lncRNA-GC1 levels and total lncRNA-GC1 levels in patients with GC (n = 15). The results are presented as the mean ± SD.

eTable 1. Clinical characteristics of individuals in test, verification and supplemental phases.

Characteristics	Test phase (n=164)		Verification phase (n=622)					Supplemental phase (n=40)
	GC (n=96)	HD (n=68)	GC (n=386)	IM (n=48)	CAG (n=37)	HD with HP^+ (n=64)	HD with HP^- (n=87)	GC (n=40)
Gender								
Male	58	48	228	31	26	42	51	24
Female	38	20	158	17	11	22	36	16
Age (years)								
Median (range)	56 (28-74)	58 (34-81)	61 (29-79)	55 (37-66)	54 (35-72)	58 (36-82)	59 (34-78)	57 (34-76)
T stage (%)		/		/	/	/	/	
T_1	19		92					7
T_2	20		87					7
T_3	29		138					19
T_4	28		69					7
N stage (%)		/		/	/	/	/	
N_0	28		81					7
N_1	36		124					13
N_2	24		138					14
N_3	8		43					6
Clinical stage (%)	17	/	85	/	/	/	/	3
I	21		94					10
II	49		167					23

© 2020 Guo X et al. *JAMA Surg.*

III<br>IV	9		40					4
Pathological grading<br>G_1<br>G_2<br>G_3	17<br>34<br>45	/	74<br>106<br>206	/	/	/	/	7<br>12<br>21
Lauren's classification<br>Intestinal<br>diffuse	66<br>30	/	307<br>79	/	/	/	/	23<br>17

GC: gastric cancer; HD: healthy donor; CAG: chronic atrophic gastritis; IM: Intestinal metaplasia; HP^+: positive Helicobacter pylori infection; HD^-: Healthy donor with negative Helicobacter pylori infection. GC-pre/post: gastric cancer-preoperative and postoperative.

eTable 2: Results of ROC curves for circulating lncRNA-GC1, CEA, CA72-4 and CA19-9.

Variables	AUC	95% CI	Sensitivity	Specificity
**Test phase**				
GC vs. HD				

© 2020 Guo X et al. *JAMA Surg.*

lncRNA-GC1	0.8905	0.8371-0.9438	88.24%	82.29%
CEA	0.5987	0.5121-0.6854	66.18%	56.25%
CA72-4	0.6816	0.6013-0.7619	63.24%	61.46%
CA19-9	0.6482	0.5603-0.7361	76.47%	63.54%
**Verification phase**				
GC vs. HD				
lncRNA-GC1	0.8977	0.8646-0.9307	84.77%	84.97%
CEA	0.6374	0.5861-0.6888	70.20%	52.07%
CA72-4	0.7288	0.6757-0.7819	78.81%	66.58%
CA19-9	0.6021	0.5392-0.6649	60.26%	69.69%
GC vs. CAG				
lncRNA-GC1	0.8491	0.7633-0.9349	89.19%	87.82%
CEA	0.5763	0.4667-0.6860	64.86%	55.70%
CA72-4	0.6103	0.5158-0.7048	67.57%	47.41%
CA19-9	0.5810	0.4988-0.6631	72.97%	56.22%
GC vs. IM				
lncRNA-GC1	0.8599	0.8197-0.9002	89.58%	80.83%
CEA	0.5794	0.4951-0.6637	66.67%	47.93%
CA72-4	0.6111	0.5303-0.6919	62.50%	50.00%
CA19-9	0.5221	0.4536-0.5905	47.92%	51.04%
EGC vs. HD				
lncRNA-GC1	0.8608	0.8188-0.9028	88.74%	79.89%
CEA	0.5950	0.5340-0.6559	68.21%	46.37%
CA72-4	0.6018	0.5404-0.6633	74.17%	50.84%
CA19-9	0.5124	0.4499-0.5750	60.26%	44.69%
EGC vs. CAG				
lncRNA-GC1	0.8841	0.8318-0.9364	91.89%	82.12%
CEA	0.5625	0.4691-0.6559	62.16%	43.58%
CA72-4	0.6052	0.5170-0.6935	70.27%	50.28%

© 2020 Guo X et al. *JAMA Surg.*

CA19-9	0.5635	0.4742-0.6528	59.46%	54.19%
EGC vs. IM				
lncRNA-GC1	0.8853	08333-0.9373	81.25%	87.71%
CEA	0.5868	0.4930-0.6807	54.17%	51.40%
CA72-4	0.6180	0.5229-0.7132	60.42%	55.87%
CA19-9	0.5435	0.4477-0.6392	54.17%	54.75%
**Test + Verification phases**				
EGC vs. HD				
lncRNA-GC1	0.8860	0.8487-0.9234	87.21%	87.10%
CEA	0.6168	0.5642-0.6694	63.47%	53.46%
CA72-4	0.6216	0.5685-0.6746	76.26%	48.39%
CA19-9	0.5497	0.4953-0.6041	52.05%	50.23%
EGC vs. CAG				
lncRNA-GC1	0.8688	0.7778-0.9598	89.19%	83.41%
CEA	0.5479	0.4307-0.6651	56.76%	55.76%
CA72-4	0.54.47	0.4325-0.6568	62.16%	46.54%
CA19-9	0.57.12	0.47.48-0.6676	59.46%	52.07%
EGC vs. IM				
lncRNA-GC1	0.8765	0.7975-0.9556	85.42%	87.10%
CEA	0.5456	0.4512-0.6399	60.42%	47.47%
CA72-4	0.5482	0.4515-0.6449	56.25%	50.69%
CA19-9	0.5241	0.4299-0.6183	52.08%	48.85%
EGC (negative) vs. HD				
lncRNA-GC1	0.9023	0.8658-0.9388	91.78%	85.16%

AUC: area under curve; CI: confidence interval; GC: gastric cancer; HD: healthy donor; CAG: chronic atrophic gastritis; IM: intestinal metaplasia; EGC: early gastric cancer; EGC (negative): GC patients with negative status of CEA, CA72-4 and CA19-9.

© 2020 Guo X et al. *JAMA Surg.*

eMethods 1. Serum and tissue samples collection.

We enrolled 96 consecutive patients with GC and 68 HDs in the test phase from December 2016 to June 2017, another 386 consecutive ones with GC, 37 ones with CAG, 48 ones with IM, 64 HD^{+}s and 87 HD^{-}s in the verification phase from August 2017 to September 2018, and 40 ones with GC who underwent gastrectomy in the supplemental phase from November 2018 to February 2019 at the Chinese PLA General Hospital. After surgery, patients with GC were all diagnosed with adenocarcinoma, which was confirmed by two pathologists. Patients previously treated with chemoradiotherapy, cytotoxic therapy, or targeted therapies were excluded. The clinical stage was determined according to the 8th AJCC (American Joint Committee on Cancer) TNM (tumor-node-metastasis) staging system[17]. Pathological grading was determined according to the World Health Organization's criteria[18]. EGC was defined as stages I and II, and advanced GC was defined as stages III and IV. Patients with chronic atrophic gastritis (CAG) and patients with intestinal metaplasia (IM) were diagnosed using endoscopy and confirmed by biopsy. Helicobacter pylori (HP) infection was confirmed using the C13 or C14 breath test. HDs were recruited from

people who underwent routine health examinations at the Chinese PLA General Hospital. The HDs had not detectable symptoms of GC, other gastric disease, or abnormal levels of tumor markers.

Blood samples were collected according to standard protocols. Briefly, 20 ml of venous blood was drawn into EDTA-coated vacuum tubes for 30 min and centrifuged at 3000 rpm for 15 min at room temperature. The serum samples were subsequently labeled with a unique identifier and stored at –80 °C.

To compare the parallel expression levels of lncRNA-GC1 in the supplemental phase, RT-PCR was used to analyze tissues, cells, and exosomes in GC samples and gastric cancer cell lines (GGCs). Samples acquired from GC patients who underwent gastrectomy were immediately frozen in liquid nitrogen for 1 h and stored at –80 °C.

eMethods 2: Cell culture

GC cell lines (MGC-803, SGC-7901, MKN-28, MKN-45, AGS, BGC-823, HGC-27, KATO III, HS-746T, and SNU-5) were obtained from the ATCC (American Type Culture Collection). The immortalized human gastric epithelial cell line GES-1 and

© 2020 Guo X et al. *JAMA Surg.*

primary gastric epithelial cells (PGECs) were obtained from the Chinese Academy of Sciences (http://www.cellbiologics.net). GC cell lines were cultured in DMEM-high-glucose medium with 10% fetal bovine serum (Gibco, USA). GES-1 cells and PGECs were cultured in complete epithelial cell medium as previously reported.

eMethods 3: Identifications of exosomes.

The identification of particles as exosomes was confirmed using transmission electron microscopy (TEM), and their size distributions were determined using a NanoSight (NS300, UK). For western blotting, proteins were extracted from exosomes using Radio Immunoprecipitation Assay (RIPA) buffer and re-extracted with sodium dodecyl sulfate (SDS) before polyacrylamide gel electrophoresis. Proteins were electrophoretically transferred to a polyvinylidene fluoride (PVDF) membrane, which was incubated with the primary antibodies as follows: anti-CD9 (1:1000; Abcam, UK), anti-CD63 (1:2000; Abcam, UK), and anti-tubulin (1:3000; Abcam, UK) at 4 °C overnight for at ≥8 h). Chemiluminescence assays were performed to visualize the bands, which were quantified using Image J software.

© 2020 Guo X et al. *JAMA Surg.*

eMethods 4: Real-time PCR.

An MMLV cDNA Kit was used to synthesize cDNA from 30 ng of exosomal RNA or 500 ng or circulating RNA (Takara, Japan). A synthetic λ polyA+ RNA (Takara) was used to spike samples as an exogenous reference. Amplifications began with denaturation at 95 °C for 5 min, followed by 40 cycles of amplification at 95 °C for 10 s, and 60 °C for 30 s. The relative levels of lncRNA-GC1 of tissues, exosomes, and plasma were normalized to that of the spiked λ polyA+ RNA using the 2ΔΔCt method. The primers used to amplify lncRNA-GC1 were obtained from Sangon Biotech are as follows:

sense: TGGGGTAACTTAGCAGTTTCAAT,

antisense: GGCAAGCAGTAATCTTACATGACAC.

© 2020 Guo X et al. *JAMA Surg.*
